# Mapping global inequities in telemedicine implementation: An umbrella review of barriers and facilitators

**DOI:** 10.1371/journal.pone.0351885

**Published:** 2026-07-13

**Authors:** Angelo Capodici, Alessandro Filippeschi, Francesca Noci, Alessandra Michelucci, Safae El Motarajji, Vera Benedetto, Andrea Vandelli, Claudio Passino, Michele Emdin, Sabina Nuti, Carlo Alberto Avizzano, Nicola Bellè, Alberto Giannoni

**Affiliations:** 1 Interdisciplinary Research Center for Health Science, Sant’Anna School of Advanced Studies, Pisa, Italy; 2 Institute of Mechanical Intelligence, Sant’Anna School of Advanced Studies, Pisa, Italy; 3 Department of Excellence in Robotics and AI, Sant’Anna School of Advanced Studies, Pisa, Italy; 4 Department of Public Health and Pediatrics, University of Torino, Turin, Italy; 5 Department of Obstetrics and Gynecology, University Hospital “Degli Infermi”, Ponderano, Italy; 6 Management and Healthcare Laboratory, Institute of Management, Sant’Anna School of Advanced Studies, Pisa, Italy; 7 Cardiology and Cardiovascular Medicine Department, Fondazione Monasterio, Pisa, Italy; Marwadi University, INDIA

## Abstract

Telemedicine expanded rapidly during the COVID-19 pandemic, yet its diffusion has remained uneven across health systems. Whether implementation challenges differ systematically across economic contexts, and what implications these differences hold for global digital health equity, has not been comprehensively characterized. This study presents an umbrella review (PROSPERO CRD42024615998) of systematic reviews and meta-analyses indexed in PubMed and Scopus through December 2025 that reported barriers and/or facilitators to telemedicine implementation. Methodological quality was appraised using R-AMSTAR. Reported items were extracted verbatim, embedded with the Universal Sentence Encoder, and grouped into thematic clusters using HDBSCAN density-based clustering, with parameters optimized by SLSQP for cluster cohesion (median intra-cluster similarity ≥ 0.5). Analyses were stratified by World Bank country income group to characterize context-specific implementation profiles, identify evidence gaps, and detect orphaned barriers, defined as challenges lacking any corresponding documented facilitator. A total of 161 systematic reviews were included, yielding 1,333 barriers and 504 facilitators (corresponding to a barrier-to-facilitator ratio of 2.6:1). Across all settings, the most frequently reported barriers were high costs (n = 106), technical issues (n = 94), and training and knowledge (n = 91). Thematic profiles differed markedly by income group: high-income countries reported predominantly second-generation challenges centered on workflow integration, interoperability, and user experience, whereas lower-middle-income countries reported foundational barriers centered on infrastructures and costs. Evidence produced from low-income countries was entirely absent. Several high-frequency barriers lacked corresponding facilitator clusters, indicating challenges for which the published literature provides no documented solutions. The global telemedicine evidence base is itself inequitably distributed and risks reinforcing the disparities it is intended to reduce. High-income settings require implementation research focused on human and organizational factors, whereas lower-income settings require foundational research and investment in basic infrastructures before workflow-level optimization becomes meaningful. Without context-specific, equity-oriented strategies, telemedicine risks widening rather than narrowing the global digital health divide.

## 1. Introduction

Few healthcare innovations have scaled as rapidly, or as unevenly, as telemedicine during the COVID-19 pandemic. Adoption rates that would otherwise have taken decades unfolded in weeks, with virtual consultations more than doubling in some healthcare systems within months [1]. Yet four years later, a paradox has emerged: despite unprecedented investment and proven technical feasibility, the global diffusion of telemedicine remains uneven [2]. It is plausible that the digital divide extends beyond platform usability; preliminary observations suggest that while high-income settings refine clinical workflows, lower-resource regions may struggle with foundational deficits like limited broadband [3] and unreliable electricity [4]. Understanding the true extent of these potential asymmetries is a fundamental step in addressing broader challenges to global health equity and Universal Health Coverage.

The promise of telemedicine as a “great equalizer” in healthcare access [5] confronts an uncomfortable reality: digital health interventions may be widening the very disparities they aim to address. Notably, it is estimated that 37% of all people globally remain offline [6], predominantly in regions with the greatest healthcare needs. When telemedicine strategies designed for resource-rich settings are exported without adaptation, they predictably fail. Despite this, the global health community still lacks a systematic, comparative understanding of which implementation challenges arise in which contexts, and why these challenges differ across health systems.

This evidence gap carries tangible consequences: misaligned investments waste scarce resources, failed deployments erode institutional and patient trust, and missed opportunities translate into avoidable health losses [7]. Implementation science has, in recent years, produced a substantial body of systematic reviews examining barriers to and facilitators of telemedicine adoption across diverse clinical specialties and care settings. However, these reviews remain scattered across disease areas, geographical regions, and organizational contexts, with limited cross-referencing or thematic integration. As a result, their findings have only limited utility for strategic decision-making at the level of national digital health policy or international health governance, where comparative and equity-sensitive evidence is most needed.

Previous attempts to synthesize narrative evidence have relied on manual categorization of barriers and facilitators, an approach that limits reproducibility, and struggles to handle the volume of available evidence [8–10]. More critically, these syntheses have failed to systematically examine how implementation challenges differ across economic contexts, treating telemedicine implementation as if the barriers faced by a tertiary hospital in a high-income country are comparable to those in a primary clinic in a low-income one. This oversight reflects a broader epistemic bias in global health, where evidence generation and policy design remain concentrated in high-income contexts.

Against this backdrop, the present umbrella review is guided by a single overarching question: how are reported barriers and facilitators to telemedicine implementation distributed across global economic contexts, and what does this distribution reveal about equity in the digital health evidence base?

To address this question, this study pursues four interrelated objectives. First, it synthesizes the existing body of systematic reviews and meta-analyses on telemedicine implementation by identifying and aggregating reported barriers and facilitators across heterogeneous clinical, geographical, and organizational settings. Second, it classifies these barriers and facilitators into coherent thematic clusters. Third, it compares the frequency, type, and thematic composition of barriers and facilitators across World Bank income groups to characterize context-specific implementation profiles. Finally, it identifies structural evidence gaps, including under-represented regions, “orphaned” barriers lacking documented facilitators, and asymmetries in the barrier-to-facilitator ratio, that may perpetuate global digital-health inequities and that should inform future implementation research and policy. Taken together, these objectives are intended to provide policymakers, funders, and researchers with an evidence-based, equity-sensitive framework for context-appropriate telemedicine investment.

As nations commit billions to digital health infrastructure and the World Health Organization develops its Global Strategy on Digital Health [11], understanding these implementation disparities is essential to ensuring that telemedicine fulfills its promise of expanding, rather than restricting, global healthcare access. By mapping these disparities through a reproducible computational approach, we aim to inform global health governance with evidence that is both scientifically rigorous and equity-sensitive.

## 2. Methods

A literature search was conducted on November 11, 2024, and on December 14, 2025 using PubMed and Scopus to identify systematic reviews and meta-analyses addressing telemedicine and related digital health interventions. This review was registered on PROSPERO (Record ID: CRD42024615998). The PRISMA Checklist can be found in the supplemental material.

Ethics Approval: Not applicable, as this umbrella review is a synthesis of previously published studies and did not involve direct contact with human participants.

The search strings combined a wide array of telemedicine-related terms, including

- On PubMed

(telemedicine OR telehealth OR telecare OR mobile health OR untact health OR mhealth OR m-health OR e-health OR ehealth OR digital health OR virtual care OR virtual consultation OR telenursing OR teleconsultation OR e-consultation OR econsult* OR e-medicine OR emedicine OR telemonitoring OR telepractice) AND (adoption OR implement* OR uptake OR accept* OR integrat* OR diffus* OR utiliz* OR utilis* OR deploy* OR embrac*)

AND (barrier* OR obstacle* OR challenge* OR hindrance* OR limitation* OR difficult* OR resist* OR constraint* OR deterrent* OR impediment* OR reluctan* OR inhibit* OR shortcoming* OR drawback* OR bottleneck* OR weakness* OR “stumbling block” OR hurdle* OR burden* OR deficiency OR flaw* OR opposition OR friction)

- On Scopus

(TITLE-ABS-KEY (telemedicine OR telehealth OR telecare OR “mobile health” OR “untact health” OR mhealth OR “m-health” OR ehealth OR “e-health” OR “digital health” OR “virtual care” OR “virtual consultation” OR telenursing OR teleconsultation OR econsult* OR “e-consultation” OR emedicine OR “e-medicine” OR telemonitoring OR telepractice)) AND (TITLE-ABS-KEY (adoption OR implement* OR uptake OR accept* OR integrat* OR diffus* OR utiliz* OR utilis* OR deploy* OR embrac*)) AND (TITLE-ABS-KEY (barrier* OR obstacle* OR challenge* OR hindrance* OR limitation* OR difficult* OR resist* OR constraint* OR deterrent* OR impediment* OR reluctan* OR inhibit* OR shortcoming* OR drawback* OR bottleneck* OR weakness* OR “stumbling block” OR hurdle* OR burden* OR deficiency OR flaw* OR opposition OR friction)) AND (LIMIT-TO (DOCTYPE, “re”))

The retrieved studies were screened blindly first by title by four authors: AC, FN, VB, AV. The remainder was screened first by abstract and then by full-text by two authors: AC, FN. In instances of disagreement, discussion was attempted first, and if a consensus was not reached a third, independent, tie-breaker was consulted (AG). Methodological quality was assessed independently and blindly by four raters (AC, FN, AM, SEM) using the R-AMSTAR tool [12].

### 2.1. Eligibility Criteria

Eligible studies had no language restriction, had to involve human subjects, and assess telemedicine interventions by exploring barriers and/or facilitators to telemedicine implementation.

Only systematic reviews and meta-analyses were considered eligible for the Umbrella Review. Studies were included if they reported on barriers or facilitators to telemedicine adoption, with the main outcome being the identification and/or quantification of these factors. Eligible reviews could present findings either through effect measures such as relative risks, odds ratios, and risk differences or via narrative synthesis.

### 2.2. Data extraction

Data extraction was performed by four independent reviewers (AC, FN, AM, SEM) using a predesigned Excel spreadsheet. Extracted data encompassed: author, publication year, journal, DOI, study design, country of origin (operationalized as the country of the first author’s first listed affiliation) and its income level as defined by the World Bank [13], type of intervention, barriers and facilitators identified, alongside the quantitative and qualitative methods employed. Demographic and population-specific data were harmonized to include sample size, population type (e.g., disease focus), ethnicity, race, and age. Finally, the setting of the study was analyzed.

Barriers and facilitators were recorded verbatim from the original reviews when reported in a concise format. In cases where items were presented in a dialogic or descriptive manner, they were initially transcribed as stated and subsequently standardized through consensus by two independent reviewers (AC, FN) to ensure consistency in terminology and categorization.

### 2.3. Data analysis

The analysis employed a data-driven pipeline to identify thematic clusters of barriers and facilitators. The process began with text preprocessing, where entries were normalized by converting them to lowercase, removing punctuation, and standardizing domain-specific terms through a combination of exact and fuzzy matching [14]. After this, the text was lemmatized [15], and stripped of stopwords. Following this preparation, the cleaned text was converted into numerical vectors using the Universal Sentence Encoder to capture the semantic meaning of each phrase. A density-based algorithm, HDBSCAN [16,17] was then used to group the embedded text into semantically coherent clusters. This method excels at finding natural groupings and identifying outliers (noise), which ensures high intra-cluster similarity (ICS). An optimization procedure, implemented using the SLSQP algorithm [18], fine-tuned the clustering parameters to maximize the number of included items while ensuring a high median ICS (≥0.5), and a second clustering phase was run on the “noise” data to recover additional themes. Finally, to identify thematic differences across socioeconomic strata, the entire analysis was repeated on data subsets stratified by country income level. All analyses were implemented in Python, and the full code, as well as all data, is available for replication at: https://github.com/AlessandroFilippeschi/Umbrella_review_Barriers_Facilitators. Further details can be found in **Supporting Information S1 File.**

## 3. Results

**Fig 1:**
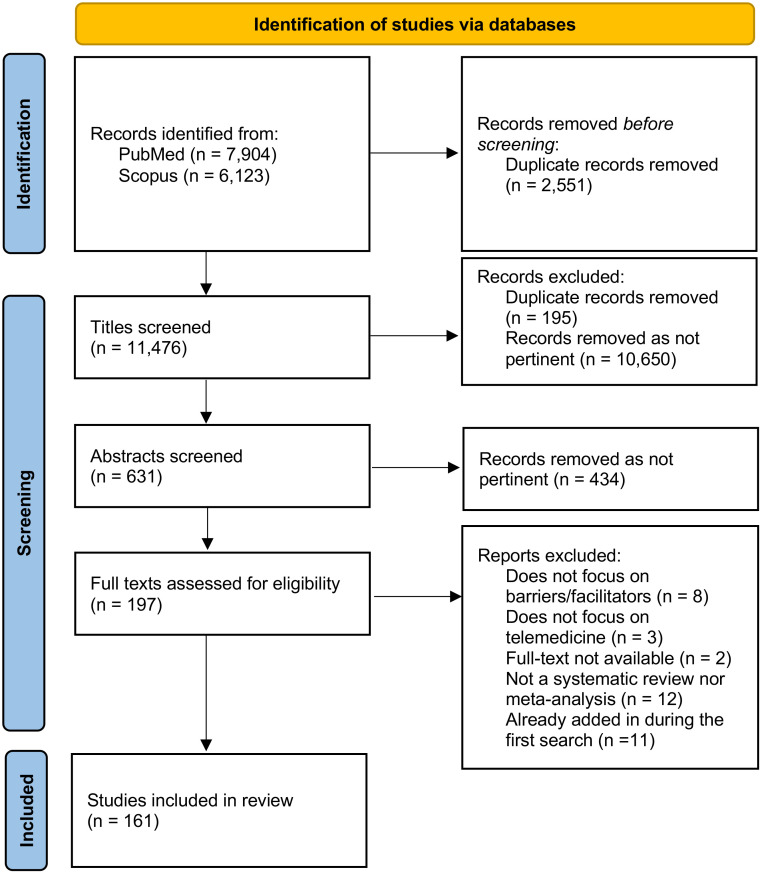
PRISMA FlowChart of included studies.

The systematic search identified 14,027 articles, which after duplicate removal and all screening phases yielded 161 systematic reviews [19–179] meeting inclusion criteria (Fig 1, Table 1).

**Table 1:** Included studies, in chronological order.

*Title*	*Author*	*Year*	*Country*	*R-AMSTAR Rate %*
*A review on remote monitoring technology applied to implantable electronic cardiovascular devices*	Costa et al.	2010	Portugal	48
*Barriers and facilitators that affect public engagement with eHealth services*	Hardiker NR et al.	2010	UK	39
*Staff acceptance of tele-ICU coverage: a systematic review*	Young LB et al.	2011	USA	61
*Telemedicine and plastic surgery: a review of its applications, limitations and legal pitfalls*	Gardiner S et al.	2012	UK	32
*Telemedicine across borders: a systematic review of factors that hinder or support implementation*	Saliba V et al.	2012	UK	73
*Implementation factors and their effect on e-Health service adoption in rural communities: a systematic literature review*	Hage et al.	2013	Netherlands	66
*Facilitators and barriers to the adoption of telehealth in older adults: an integrative review*	Foster MV et al.	2014	USA	50
*Home telehealth uptake and continued use among heart failure and chronic obstructive pulmonary disease patients: a systematic review*	Gorst et al.	2014	UK	73
*Barriers for delivering telehealth in rural australia: a review based on Australian trials and studies*	Jang-Jaccard et al.	2014	Australia	45
*Acceptability of Interventions Delivered Online and Through Mobile Phones for People Who Experience Severe Mental Health Problems: A Systematic Review*	Berry et al.	2016	UK	57
*Barriers and Facilitators for Sustainability of Tele-Homecare Programs: A Systematic Review*	Radhakrishnan et al.	2016	USA	64
*Factors that influence the implementation of e-health: a systematic review of systematic reviews (an update)*	Ross J et al.	2016	UK	61
*A mixed methods systematic review of success factors of mhealth and telehealth for maternal health in Sub-Saharan Africa*	Ag Ahmed et al.	2017	Canada	80
*Barriers to Remote Health Interventions for Type 2 Diabetes: A Systematic Review and Proposed Classification Scheme*	Alvarado et al.	2017	USA	70
*A Systematic Review of Wearable Patient Monitoring Systems – Current Challenges and Opportunities for Clinical Adoption*	Baig et al.	2017	New Zealand	50
*Experiences of community-dwelling older adults with the use of telecare in home care services: a qualitative systematic review*	Karlsen C et al.	2017	Denmark	59
*Barriers and opportunities to implementation of sustainable e-Health programmes in Uganda: A literature review*	Kiberu et al.	2017	South Africa	45
*A Systematic Review of the Implementation Challenges of Telemedicine Systems in Ambulances*	Rogers H et al.	2017	USA	43
*A Systematic Review of the Use of Telemedicine in Plastic and Reconstructive Surgery and Dermatology*	Vyas et al.	2017	USA	55
*Real-Time Remote Health-Monitoring Systems in a Medical Centre: A Review of the Provision of Healthcare Services-Based Body Sensor Information, Open Challenges and Methodological Aspects*	Albahri OS et al.	2018	Malaysia	36
*Study of challenges to utilise mobile-based health care monitoring systems: A descriptive literature review*	Baniasadi T et al.	2018	Iran	57
*Nursing professionals’ experiences of the facilitators and barriers to the use of telehealth applications: a systematic review of qualitative studies*	Koivunen et al.	2018	Finland	66
*Factors influencing the adoption of telemedicine for treatment of military veterans with post-traumatic stress disorder*	Kruse et al.	2018	USA	59
*Consumer perspectives on mHealth for weight loss: a review of qualitative studies*	Lyzwinski et al.	2018	Australia	52
*Evaluating barriers to adopting telemedicine worldwide: A systematic review*	Scott Kruse et al.	2018	USA	68
*Barriers to and Facilitators of Engagement With Remote Measurement Technology for Managing Health: Systematic Review and Content Analysis of Findings*	Simblett et al.	2018	UK	77
*Systematic review of lessons learned from delivering tele-therapy to veterans with post-traumatic stress disorder*	Turgoose D et al.	2018	UK	57
*Factors affecting implementation of digital health interventions for people with psychosis or bipolar disorder, and their family and friends: a systematic review*	Aref-Adib et al.	2019	UK	64
*A comparative review of mobile health and electronic health utilization in sub-Saharan African countries*	Bervell et al.	2019	Ghana	39
*What Works and What Doesn’t Work? A Systematic Review of Digital Mental Health Interventions for Depression and Anxiety in Young People*	Garrido S et al.	2019	Australia	50
*Geriatric Telepsychiatry: Systematic Review and Policy Considerations*	Gentry et al.	2019	USA	57
*Barriers to the Use of Mobile Health in Improving Health Outcomes in Developing Countries: Systematic Review*	Kruse et al.	2019	USA	64
*A Systematic Review of Nurses’ Perspectives Toward the Telemedicine Intensive Care Unit: A Basis for Supporting Its Future Implementation in China?*	Li et al.	2019	China	64
*Barriers and Facilitators to the Implementation of eHealth Services: Systematic Literature Analysis*	Schreiweis et al.	2019	Germany	45
*Patients’ Perceptions of mHealth Apps: Meta-Ethnographic Review of Qualitative Studies*	Vo V et al.	2019	USA	39
*What Are the Challenges and Facilitators for Implementing a 24-Hour Telephone Cancer Service in Qatar? A Literature Review*	Zawahreh A et al.	2019	Qatar	30
*Telemedicine in Middle Eastern countries: Progress, barriers, and policy recommendations*	Al-Samarraie H et al.	2020	UK	73
*Barriers and Facilitators That Influence Telemedicine-Based, Real-Time, Online Consultation at Patients’ Homes: Systematic Literature Review*	Almathami et al.	2020	Australia	64
*A systematic review of providers’ attitudes toward telemental health via videoconferencing*	Connolly et al.	2020	USA	48
*A Systematic Review of Pediatric Telediabetes Service Models*	De Guzman et al.	2020	Australia	64
*A Framework-Driven Systematic Review of the Barriers and Facilitators to Teledermatology Implementation*	Dovigi et al.	2020	USA	70
*Facilitators and barriers of using digital technology for the management of diabetic foot ulcers: A qualitative systematic review*	Foong et al.	2020	Singapore	75
*The current use of telehealth in ALS care and the barriers to and facilitators of implementation: a systematic review*	Helleman J et al.	2020	Netherlands	52
*Utilization Barriers and Medical Outcomes Commensurate With the Use of Telehealth Among Older Adults: Systematic Review*	Kruse C et al.	2020	USA	75
*Challenges to the Emergence of Telerehabilitation in a Developing Country: A Systematic Review*	Leochico et al.	2020	Philippines	64
*Engaging Children and Young People in Digital Mental Health Interventions: Systematic Review of Modes of Delivery, Facilitators, and Barriers*	Liverpool et al.	2020	UK	61
*Barriers and facilitators of videoconferencing psychotherapy implementation in veteran mental health care environments: a systematic review*	Muir et al.	2020	Australia	66
*Telemedicine Practice: Review of the Current Ethical and Legal Challenges*	Nittari G et al.	2020	Italy	45
*An exploration of usability issues in telecare monitoring systems and possible solutions: a systematic literature review*	Saeed et al.	2020	Sweden	64
*Barriers to Sustainable Telemedicine Implementation in Ethiopia: A Systematic Review*	Sagaro et al.	2020	Italy	68
*Implementation of Telerehabilitation Interventions for the Self-Management of Cardiovascular Disease: Systematic Review*	Subedi N et al.	2020	Australia	52
*Barriers and facilitators to patient uptake and utilisation of digital interventions for the self-management of low back pain: a systematic review of qualitative studies*	Svendsen et al.	2020	Denmark	70
*Barriers to and Facilitators of User Engagement With Digital Mental Health Interventions: Systematic Review*	Borghouts et al.	2021	USA	73
*The efficacy, challenges, and facilitators of telemedicine in post-treatment cancer survivorship care: an overview of systematic reviews*	Chan RJ et al.	2021	Australia	70
*Telemedicine use in Sub-Saharan Africa: Barriers and policy recommendations for Covid-19 and beyond*	Dodoo JE et al.	2021	Ghana	50
*A Systematic Review of Telemedicine for Older Adults With Dementia During COVID-19: An Alternative to In-person Health Services?*	Elbaz et al.	2021	Canada	55
*“Wearables only work on patients that wear them”: Barriers and facilitators to the adoption of wearable cardiac monitoring technologies*	Ferguson et al.	2021	Australia	55
*Telehealth-Based Services During the COVID-19 Pandemic: A Systematic Review of Features and Challenges*	Khoshrounejad F et al.	2021	Iran	59
*eHealth in Geriatric Rehabilitation: Systematic Review of Effectiveness, Feasibility, and Usability*	Kraaijkamp et al.	2021	Netherlands	84
*Older Adults’ Experiences With Using Wearable Devices: Qualitative Systematic Review and Meta-synthesis*	Moore K et al.	2021	Ireland	57
*A Review of Patient Satisfaction and Experience with Telemedicine: A Virtual Solution During and Beyond COVID-19 Pandemic*	Nanda et al.	2021	India	43
*Healthcare stakeholders’ perceptions and experiences of factors affecting the implementation of critical care telemedicine (CCT): qualitative evidence synthesis*	Xyrichis et al.	2021	UK	82
*Telemedicine and Dementia Care: A Systematic Review of Barriers and Facilitators*	Yi JS et al.	2021	USA	66
*Objectives, Outcomes, Facilitators, and Barriers of Telemedicine Systems for Patients with Alzheimer’s Disease and their Caregivers and Care Providers: A Systematic Review*	Amiri et al.	2022	Iran	66
*Meaningful patient and public involvement in digital health innovation, implementation and evaluation: A systematic review*	Baines R et al.	2022	UK	45
*Promotors and barriers to the implementation and adoption of assistive technology and telecare for people with dementia and their caregivers: a systematic review of the literature*	Boyle LD et al.	2022	Norway	68
*Optimising implementation of telehealth in oncology: A systematic review examining barriers and enablers using the RE-AIM planning and evaluation framework*	Bu et al.	2022	Australia	59
*Analysis of barriers and benefits associated with e-health technology applications*	Calegari et al.	2022	Brazil	43
*At my own pace, space, and place: A systematic review of qualitative studies of enablers and barriers to telehealth interventions for people with chronic pain*	Fernandes et al.	2022	Brazil	64
*Challenges of Telemedicine during the COVID-19 pandemic: a systematic review*	Ftouni et al.	2022	Lebanon	75
*Barriers and Supports in eHealth Implementation among People with Chronic Cardiovascular Ailments: Integrative Review*	Herrera et al.	2022	Chile	52
*A mixed methods systematic review of digital interventions to support the psychological health and well-being of people living with dermatological conditions*	Hewitt et al.	2022	UK	82
*Barriers and facilitators for the sustainability of digital health interventions in low and middle-income countries: A systematic review*	Kaboré et al.	2022	Burkina Faso	68
*Facilitators and Barriers to the Adoption of Telemedicine During the First Year of COVID-19: Systematic Review*	Kruse et al.	2022	USA	68
*Enablers and Challenges for E-Health Services: A Systematic Literature Review*	Kumari et al.	2022	India	41
*eHealth in TB clinical management*	Margineanu et al.	2022	Romania	84
*Applications and Current Medico-Legal Challenges of Telemedicine in Ophthalmology*	Mazzuca et al.	2022	Italy	50
*Patients’ acceptance of video consultations in the mental health services: A systematic review and synthesis of qualitative research*	Moeller et al.	2022	Denmark	66
*Benefits and challenges of telepsychiatry services in SouthEast Asian nations during the COVID-19 era: An integrative review*	Narvaez	2022	Philippines	50
*Integrating Telepsychiatry Based Care in Rural Acute Community Mental Health Services? A Systematic Literature Review*	Noble et al.	2022	Australia	57
*Barriers to and Facilitators of Using eHealth to Support Gestational Diabetes Mellitus Self-management: Systematic Literature Review of Perceptions of Health Care Professionals and Women With Gestational Diabetes Mellitus*	Safiee et al.	2022	UK	73
*Factors influencing the delivery of telerehabilitation for stroke: A systematic review*	Stephenson A et al.	2022	UK	61
*Using an Integrated Framework to Investigate the Facilitators and Barriers of Health Information Technology Implementation in Noncommunicable Disease Management: Systematic Review*	Sung M et al.	2022	Republic of Korea	64
*Patients’ and clinicians’ experiences of remote consultation? A narrative synthesis*	Walthall et al.	2022	UK	68
*A literature review of telemedicine in Indonesia: past, present, and future prospective; [Tinjauan Literatur Terkait Perkembangan Telemedicine di Indonesia: Dahulu, Sekarang, dan Masa Depan]*	Wijaya JH et al.	2022	Indonesia	43
*Revisión sistemática de aceptación de la tecnología digital en personas mayores. Perspectiva de los modelos TAM*	Murciano et al.	2022	Spain	57
*Older Adults’ Satisfaction with Telemedicine During the COVID-19 Pandemic: A Systematic Review*	Alsabeeha et al.	2023	United Arab Emirates	75
*Factors Influencing the Acceptance and Adoption of Mobile Health Apps by Physicians During the COVID-19 Pandemic: Systematic Review*	Alsahli et al.	2023	Australia	61
*Barriers to the implementation of virtual care programmes for patients with chronic wounds: Qualitative empirical research*	Babaei N et al.	2023	Iran	43
*Efficacy, perception, and utilization of pediatric teledermatology: A systematic review*	Burshtein J et al.	2023	USA	41
*Facilitators of and Barriers to Accessing Hospital Medical Specialty Telemedicine Consultations During the COVID-19 Pandemic: Systematic Review*	Cunha et al.	2023	Portugal	66
*What Are the Barriers to Telerehabilitation in the Treatment of Musculoskeletal Diseases?*	Franco JB et al.	2023	Brazil	75
*Health Providers’ Perceptions and Experiences of Using mHealth for Chronic Noncommunicable Diseases: Qualitative Systematic Review and Meta-Synthesis*	Gu et al.	2023	China	75
*Applications, opportunities, and challenges in using Telehealth for burn injury management: A systematic review*	Hayavi-Haghighi et al.	2023	Iran	70
*A cross-sector systematic review and synthesis of knowledge on telemedicine interventions in chronic wound management-Implications from a system perspective*	Høyland et al.	2023	Norway	61
*Analyzing the Effectiveness of mHealth to Manage Diabetes Mellitus Among Adults Over 50: A Systematic Literature Review*	Kruse et al.	2023	USA	66
*Experiences of Women With Breast Cancer Using Telehealth: A Qualitative Systematic Review*	Meneses et al.	2023	Brazil	70
*Requirements, Challenges, and Key Components to Improve Onboard Medical Care Using Maritime Telemedicine: Narrative Review*	Mohammadzadeh N et al.	2023	Iran	55
*Applications, benefits and challenges of telehealth in India during COVID-19 pandemic and beyond: a systematic review*	Rajkumar et al.	2023	India	64
*Barriers to Video Call-Based Telehealth in Allied Health Professions and Nursing: Scoping Review and Mapping Process*	Rettinger et al.	2023	Austria	59
*The State-of-the-Art of Patient Portals: Adapting to External Factors, Addressing Barriers, and Innovating*	Reynolds TL et al.	2023	USA	45
*Benefits and Challenges of Remote Patient Monitoring as Perceived by Health Care Practitioners: A Systematic Review*	Serrano et al.	2023	USA	55
*Facilitators and barriers to the adoption of mHealth apps for COVID-19 contact tracing: a systematic review of the literature*	Sujarwoto et al.	2023	Indonesia	75
*A Systematic Review of Patient-Perceived Barriers and Facilitators to the Adoption and Use of Remote Health Technology to Manage Diabetes and Cardiovascular Disease among Disproportionately Affected Populations*	Wagle et al.	2023	USA	64
*Exploring facilitators and barriers of the sustainable acceptance of e-health system solutions in Ethiopia: A systematic review*	Walle et al.	2023	Ethiopia	52
*Barriers to and Facilitators of Digital Health Among Culturally and Linguistically Diverse Populations: Qualitative Systematic Review*	Whitehead et al.	2023	Australia	73
*Experiences of remote consultation in UK primary care for patients with mental health conditions: A systematic review*	Antonio et al.	2024	UK	82
*Barriers and facilitators to the implementation of digital technologies in mental health systems: a qualitative systematic review to inform a policy framework*	Berardi et al.	2024	Australia	68
*Barriers and facilitators to health technology adoption by older adults with chronic diseases: an integrative systematic review*	Bertolazzi et al.	2024	Italy	70
*Facilitators and barriers to implementation of telemedicine in nursing homes: A qualitative systematic review and meta-aggregation*	Chua et al.	2024	Singapore	70
*Analysis of the virtual healthcare model in Latin America: a systematic review of current challenges and barriers*	De La Torre et al.	2024	Colombia	64
*Assessing Telehealth in Palliative Care: A Systematic Review of the Effectiveness and Challenges in Rural and Underserved Areas*	Ghazal KY et al.	2024	Saudi Arabia	64
*Midwives’ experience of telehealth and remote care: a systematic mixed methods review*	Golden BN et al.	2024	USA	64
*Challenges of Implementing Telemedicine Technology: A systematized Review*	Hadian et al.	2024	Iran	57
*Advancing telemedicine in cardiology: A comprehensive review of evolving practices and outcomes in a postpandemic context*	Huerne K et al.	2024	Canada	48
*Barriers to implementation of digital transformation in the Indian health sector: a systematic review*	Inampudi et al.	2024	India	73
*Understanding the Barriers and Facilitators of Digital Health Technology (DHT) Implementation in Neurological Rehabilitation: An Integrative Systematic Review*	Jarvis K et al.	2024	UK	59
*The Benefits and Challenges of Implementing Teleophthalmology in Low-Resource Settings: A Systematic Review*	Khan IA et al.	2024	India	64
*Factors Influencing the Engagement with Electronic Mental Health Technologies: A Systematic Review of Reviews*	Khosravi et al.	2024	Iran	68
*Barriers to telemedicine adoption among rural communities in developing countries: A systematic review and proposed framework*	Lestari et al.	2024	Indonesia	70
*The facilitators and barriers to implementing virtual visits in intensive care units: A mixed-methods systematic review*	Li M et al.	2024	China	66
*Insights, Advantages, and Barriers of Teledermatology vs. Face-to-Face Dermatology for the Diagnosis and Follow-Up of Non-Melanoma Skin Cancer: A Systematic Review*	Nikolakis et al.	2024	Germany	57
*Current Status of Barriers to mHealth Access Among Patients With Stroke and Steps Toward the Digital Health Era: Systematic Review*	Niyomyart et al.	2024	Thailand	64
*Facilitators of, barriers to, and preferences for e-mental health interventions for depression and anxiety in men: Metasynthesis and recommendations*	Opozda et al.	2024	Australia	70
*Implementation barriers and facilitators of remote monitoring, remote consultation and digital care platforms through the eyes of healthcare professionals: a review of reviews*	Oudbier et al.	2024	Netherlands	66
*Barriers and facilitators to acceptance and implementation of eMental-health intervention among older adults: A qualitative systematic review*	Peng et al.	2024	China	82
*A comprehensive review of mobile user interfaces in mHealth applications for elderly and the related ageing barriers*	Ramdowar et al.	2024	Mauritius	50
*Challenges and recommendations in the implementation of audiovisual telemedicine communication: a systematic review*	Ritunga et al.	2024	Indonesia	59
*Barriers and facilitators of health professionals in adopting digital health-related tools for medication appropriateness: A systematic review*	Rodrigues et al.	2024	Portugal	75
*A systematic review of synchronous telepharmacy service models for adult outpatients with cancer*	Ryan et al.	2024	Australia	70
*Acceptability and Satisfaction of Patients and Providers With Telemedicine During the COVID-19 Pandemic: A Systematic Review*	Saeed et al.	2024	India	68
*A Systematic Review of Telehealth Applications in Endocrinology*	SeyedAlinaghi et al.	2024	Iran	64
*Security and Privacy of Technologies in Health Information Systems: A Systematic Literature Review*	Shojaei P et al.	2024	Australia	55
*A systematic review of telemedicine systems use barriers: primary health care providers’ perspective*	Tabaeeian et al.	2024	Iran	48
*Factors influencing older adults’ participation in telehealth interventions for primary prevention and health promotion: A rapid review*	Turcotte S et al.	2024	Canada	52
*Barriers and Facilitators to the Implementation of Digital Health Services for People With Musculoskeletal Conditions in the Primary Health Care Setting: Systematic Review*	van Tilburg et al.	2024	Netherlands	66
*Facilitators and Barriers for Telemedicine Systems in India from Multiple Stakeholder Perspectives and Settings: A Systematic Review*	Venkataraman et al.	2024	India	66
*Facilitators, barriers, and guidance to successful implementation of multidisciplinary transitional care interventions: A qualitative systematic review using the consolidated framework for implementation research*	Collet et al.	2024	Netherlands	73
*A Systematic Review of Publications on Perceptions and Management of Chronic Medical Conditions Using Telemedicine Remote Consultations by Primary Healthcare Professionals April 2020 to December 2021 During the COVID-19 Pandemic*	Ahmed et al.	2024	UK	64
*Barriers and facilitators for the use of telehealth by healthcare providers in India-A systematic review*	Sharma et al.	2024	India	64
*Telemedicine Adoption in Developing Economies: A Systematic Review on the Enablers and Barriers*	Macabato et al.	2024	Philippines	50
*Factors Influencing Telehealth Adoption in Managing Healthcare in Saudi Arabia: A Systematic Review*	Alamri et al.	2024	Saudi Arabia	59
*Barriers and Facilitators of Acceptance of Mobile Applications Regarding Health-Related Issues in the Elderly: A Systematic Review*	Miri et al.	2024	Iran	68
*Strengths, Weaknesses, Opportunities, and Threats (SWOT) of Implementing Teleconsultation: A Systematic Review*	Khoshsirat et al.	2025	Iran	57
*Factors influencing the adoption and acceptance of eHealth in Malaysia: a systematic review*	Sampa et al.	2025	Malaysia	48
*Barriers and facilitators to the use of virtual wards: A systematic review of the qualitative evidence*	Cucurachi et al.	2025	Ireland	73
*A Systematic Literature Review of Innovations, Challenges, and Future Directions in Telemonitoring and Wearable Health Technologies*	Eduard Stan et al.	2025	Italy	66
*Enablers and Barriers of Telemedicine in Indonesia: A Systematic Review*	Anandari et al.	2025	Indonesia	45
*Systematic review of barriers and facilitators to digital health technology interventions for chronic disease management in Ethiopia: Insights for implementing digital health in developing countries*	Adem et al.	2025	Ethiopia	77
*Facilitators and barriers to mobile health adoption among older adults with hip fractures: A systematic review*	Song et al.	2025	China	68
*Telehealth adoption in palliative care: a systematic review of patient barriers and facilitators*	Kirby et al.	2025	Ireland	73
*Barriers and facilitators of provision of telemedicine in Nigeria: A systematic review*	Cole et al.	2025	Nigeria	68
*Barriers to Telemedicine Establishment in Iran: A Systematic Review*	Mehrolhassani et al.	2025	Iran	80
*Patient-Related Barriers to Digital Technology Adoption in Alzheimer Disease: Systematic Review*	Panzavolta et al.	2025	Italy	70
*Acceptability, Needs, Concerns, and Barriers to Digital-Based Interventions for the Prevention of Mother-to-Child Transmission of HIV: Systematic Review and Qualitative Meta-Aggregation*	Maulana et al.	2025	Indonesia	77
*Factors Influencing Health Care Technology Acceptance in Older Adults Based on the Technology Acceptance Model and the Unified Theory of Acceptance and Use of Technology: Meta-Analysis*	Yang et al.	2025	South Korea	82
*Patient and Carer-Related Facilitators and Barriers to the Adoption of Assistive Technologies for the Care of Older Adults: Systematic Review*	Malden et al.	2025	UK	86
*Barriers to the use of telepsychiatry for the treatment of eating disorders: A systematic review and thematic synthesis*	Sapouna et al.	2025	Greece	57
*Barriers and facilitators to digital health technology adoption by older adults with chronic disease: an updated systematic review*	Hepburn et al.	2025	UK	84
*Barriers Leading to the Discontinuance of Telemedicine among Healthcare Providers: A Systematic Review; Barreras que Conducen a la Discontinuación de la Telemedicina entre los Proveedores de Atención Médica: Una Revisión Sistemática*	Bernabe et al.	2025	Philippines	36
*Systematic review of challenges of telehealth-based intervention in managing cancer pain*	Mogan et al.	2025	Malaysia	57

The geographical distribution showed stark disparities: high-income countries contributed most evidence, while lower-middle-income countries had limited representation and low-income settings produced no eligible studies.

Cluster analysis identified 1,333 barriers (ICS: 0.82) and 504 facilitators (ICS: 0.76), yielding a 2.6:1 barrier-to-facilitator ratio (Table 2). Subsequent analyses used non-reclustered data for optimal cluster consistency. Complete reclustered data and detailed cluster compositions are available in the public repository (https://github.com/AlessandroFilippeschi/Umbrella_review_Barriers_Facilitators).

**Table 2:** Summary of barriers and facilitators’ counts.

Income Level	Barriers				Facilitators			
**No Recluster**		**Recluster**		**No Recluster**		**Recluster**	
**Count**	**ICS**	**Count**	**ICS**	**Count**	**ICS**	**Count**	**ICS**
***All***	1333 [568]	0.82	1447 [454]	0.77	504 [650]	0.76	621 [533]	0.69
***High***	721 [424]	0.89	918 [227]	0.86	355 [475]	0.71	408 [422]	0.69
***Upper-Middle***	210 [278]	0.91	250 [238]	0.81	27 [167]	0.37	27 [167]	0.37
***Lower-Middle***	103 [123]	0.64	148 [78]	0.52	0 [91]	–	0 [91]	–
***Low***	0 [42]	–	0 [42]	–	0 [39]	–	0 [39]	–

* Count of items classified as “noise” are presented in square brackets.

By income level, high-income countries contributed 721 barriers and 355 facilitators; upper-middle-income countries 210 barriers and 27 facilitators; lower-middle-income countries 103 barriers with no facilitators. Low-income countries showed no clusters.

### 3.1. Thematic structure of implementation barriers

Clustering identified distinct barrier themes (Table 3, Supporting Information S2 File). The most frequent were “High Costs” (n = 106, ICS: 0.92), “Technical Issues” (n = 94, ICS: 0.99), and “Training & Knowledge” (n = 91, ICS: 0.99).

**Table 3:** Barriers and facilitator clusters, regardless of income.

Barriers			Facilitators		
**Cluster Name**	**Count**	**ICS**	**Cluster Name**	**Count**	**ICS**
High Costs	106	0.92	Connectivity & Access	25	0.64
Technical Issues	94	0.99	Training & Knowledge	22	1.00
Training & Knowledge	91	0.99	Device Availability	22	0.40
Connectivity & Access	89	0.96	Clear Instructions	19	0.57
Privacy & Security	81	0.97	Personalization Options	18	0.60
Legal & Regulatory	63	1.00	Educational Programs	18	0.71
Access & Usability	54	0.99	Perceived Ease of Use	18	0.90
Psychosocial Factors	46	1.00	Infrastructure & Social Support	16	0.79
Infrastructure Requirements	46	0.93	Motivation & Communication	15	0.94
Personal Motivation	42	0.99	Privacy & Security	14	0.99
Usability & Design	41	1.00	Financial & Team Costs	14	0.90
Digital Literacy	32	0.99	Legal & Regulatory	13	0.97
Workflow Integration & Legal	31	0.97	Technical Issues Absence	13	0.99
Language & Culture	27	1.00	Standardized Protocols	13	0.73
Psychosocial Support	27	0.97	Cost Effectiveness	13	0.45
Organizational Issues	26	1.00	Digital Literacy	13	0.85
Time & Workload	26	1.00	Staff Training & Motivation	13	0.70
Privacy Concerns	22	0.85	User-Friendly Design	13	0.81
Clinical Limitations	19	1.00	Leadership Support	11	0.78
Data Management	18	1.00	Perceived Usefulness	11	0.81

**Fig 2:**
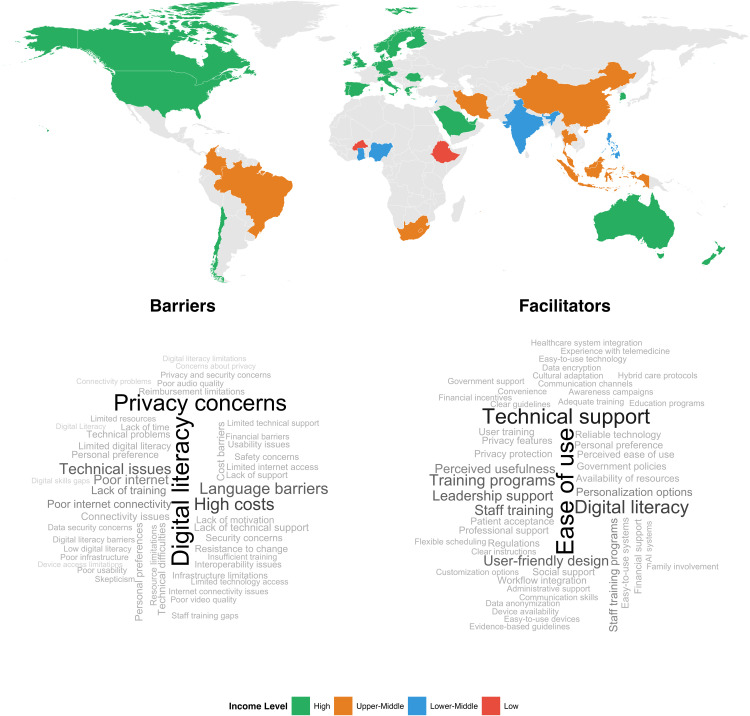
Geographic Distribution of Included Studies by Income.

**Fig 3:**
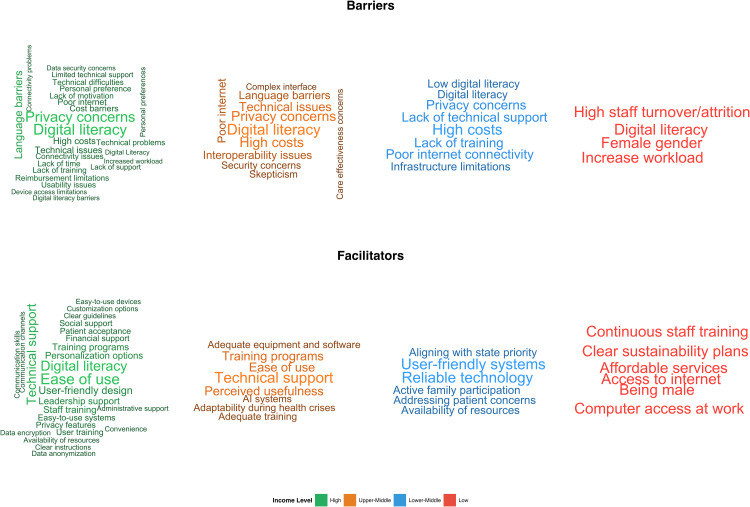
Barriers and Facilitators divided per income level.

Barrier patterns varied substantially by income level (Fig 2 and 3), with high-income countries showing diverse, specific challenges while lower-income settings displayed fewer, overlapping barrier clusters.

Table 4 presents the top 10 barriers stratified by income level, while Supporting Information S3 File provides the list of all barrier items classified in each cluster, including all the remaining not presented in the table.

**Table 4:** Barriers clusters divided by income.

***High***			***Upper Middle***			***Lower Middle***		
**Cluster Name**	**Count**	**ICS**	**Cluster Name**	**Count**	**ICS**	**Cluster Name**	**Count**	**ICS**
Connectivity & Access	82	0.73	Technical Issues	32	0.98	Training & Compliance	72	0.17
Financial & Personal Costs	64	0.94	Platform Costs	32	0.92	Infrastructure & Costs	17	0.89
Training & Knowledge	59	1.00	Connectivity & Speed	24	0.88	Connectivity & Access	14	0.86
Technical Issues	56	1.00	Privacy & Technical Issues	21	0.90			
Privacy & Security	48	1.00	Training & Relationships	21	0.84			
Access & Availability	37	0.99	Legal & Environmental	18	0.93			
Legal & Clinical Limitations	34	0.95	Workflow Integration	14	1.00			
Personal Motivation	31	1.00	Psychosocial & Legal Factors	12	0.94			
Usability & Design	31	1.00	Digital Literacy & Regulations	12	0.85			
Digital Literacy	30	0.74	Access & Usability	12	0.89			

High-income countries showed distinct clusters led by “Connectivity & Access” (n = 82, ICS: 0.73), “Financial & Personal Costs” (n = 64, ICS: 0.94), and “Training & Knowledge” (n = 59, ICS: 1.00) (**Fig 3**). Upper-middle-income countries exhibited clusters led by “Technical Issues” (n = 32, ICS: 0.98), “Platform Costs” (n = 32, ICS: 0.92), and “Connectivity & Speed” (n = 24, ICS: 0.88). Lower-middle-income countries displayed only three barrier clusters: “Training & Compliance” (n = 72, ICS: 0.17), “Infrastructure & Costs” (n = 17, ICS: 0.89), and “Connectivity & Access” (n = 14, ICS: 0.86). No barrier clusters were identified for low-income countries.

### 3.2. Thematic structure of implementation facilitators

The analysis identified facilitator clusters (Table 3, Supporting Information S2 File), led by “Connectivity & Access” (n = 25, ICS: 0.64) and “Training & Knowledge” (n = 22, ICS: 1.00).

The variation in distribution of facilitators across income levels was high as illustrated in Fig 3. Table 5 shows the top facilitators stratified per income level, while Supporting Information S4 File lists all facilitator items classified in each cluster, including all the remaining not presented in the table.

**Table 5:** Facilitator clusters divided by income.

High			Upper Middle		
Cluster Name	Count	ICS	Cluster Name	Count	ICS
Family Support & Involvement	33	0.33	User-Friendliness	19	0.16
Personalization Options	16	0.63	Training & Knowledge	8	0.57
Clear Instructions & Guidelines	15	0.64			
Training & Knowledge	14	0.99			
Connectivity & Availability	14	0.57			
Security Training Programs	14	0.52			
Motivation & Communication	13	0.93			
Team Costs & Motivation	13	0.94			
Collaboration & Assessment Tools	13	0.32			
Ease of Use	13	0.87			

High-income countries showed facilitator clusters led by “Family Support & Involvement” (n = 33, ICS: 0.33) and “Personalization Options” (n = 16, ICS: 0.63), while upper-middle-income countries had clusters led by “User-Friendliness” (n = 19, ICS: 0.16) and “Training & Knowledge” (n = 8, ICS: 0.57). Lower-middle-income and low-income countries showed no facilitator clusters, representing a complete evidence absence for these settings.

### 3.3. Implementation Gaps: Barriers Without Facilitators

Numerous high-frequency implementation challenges lack corresponding, well-documented solutions. To systematically identify these evidence gaps, **Table 6** maps each barrier against the availability of associated facilitator. This analysis reveals critical “orphaned barriers”, categorized as high-frequency obstacles without documented enablers that represent urgent priorities for implementation research (**bolded in the table**).

**Table 6:** Implementation Prioritization: Barriers and Facilitators by Frequency and Solution Availability.

Category	Barrier Cluster	N. Barrier	Corresponding Facilitator Cluster	N. Facilitator
**High Frequency Barriers WITH Corresponding Facilitators**	High Costs	106	Financial & Team Costs	14
	Technical Issues	94	Technical Issues Absence	13
	Training & Knowledge	91	Training & Knowledge	22
	Connectivity & Access	89	Connectivity & Access	25
	Privacy & Security	81	Privacy & Security	14
	Legal & Regulatory	63	Legal & Regulatory	13
	Access & Usability	54	Perceived Ease of Use	18
	Infrastructure Requirements	46	Infrastructure & Social Support	16
	Personal Motivation	42	Motivation & Communication	15
	Usability & Design	41	User-Friendly Design	13
	Digital Literacy	32	Digital Literacy	13
	Workflow Integration & Legal	31	Standardized Protocols	13
	Language & Culture	27	Personalization Options	18
**High Frequency Barriers WITHOUT Corresponding Facilitators**	**Psychosocial Factors**	**46**	.	.
	**Psychosocial Support**	**27**	.	.
	**Time & Workload**	**26**	.	.
	**Organizational Issues**	**26**	.	.
	**Privacy Concerns**	**22**	.	.
	**Clinical Limitations**	**19**	.	.
	**Data Management**	**18**	.	.

The matrix identifies Psychosocial Factors as the most critical domain, appearing as the highest-frequency barrier without documented facilitators, with 46 barrier instances and no corresponding facilitator. Psychosocial Support (n = 27), Time & Workload (n = 26), and Organizational Issues (n = 26) similarly persist without documented strategies to enhance institutional readiness or change management capacity. Privacy Concerns (n = 22), Clinical Limitations (n = 19), and Data Management (n = 18) also remain unaddressed by corresponding facilitators.

Even where facilitators are present, substantial imbalances persist. High Costs presents a concerning 7.5:1 deficit, with 106 barrier instances but only 14 facilitator instances, suggesting that financial constraints remain poorly addressed by existing support structures. The Legal and Regulatory framework presents a similarly wide imbalance with an approximate 5:1 barrier-to-facilitator ratio (63 vs. 13 instances), indicating limited progress in contexts lacking enabling legislation. Language and Cultural barriers (n = 27) show a smaller yet persistent shortfall with a 1.5:1 ratio, with 18 facilitators reflecting partial but still insufficient adaptation strategies considering the high prevalence of problems related to this issue. **S1** –**S4 Images** are presented as Supporting Information illustrating barriers and facilitators per income level.

## 4. Discussion

The COVID-19 pandemic acted as a global stress test for digital health systems, accelerating the adoption of telemedicine to a degree that few healthcare innovations have ever experienced. Four years later, this umbrella review of 161 systematic reviews allows a structured assessment of what this acceleration has produced in terms of accumulated implementation knowledge, and for whom such knowledge is available. Rather than a homogeneous global evidence base, the findings reveal a stratified landscape, in which the geography of telemedicine research closely mirrors global income distribution.

Three findings stand out and frame the analysis that follows. First, barriers (n = 1,333) outnumber facilitators (n = 504) by a ratio of 2.6 to 1, a global asymmetry that holds across income strata. Second, the thematic structure of barriers is not universal but income-contingent, with high-income countries reporting fragmented, “second-generation” challenges and lower-middle-income settings reporting miscellaneous and infrastructural ones. Third, several high-frequency barrier domains, particularly psychosocial, organizational and workload-related, exist as “orphaned” clusters, lacking any corresponding facilitator in the literature. Each of these findings is examined in detail in the following sections, before their combined implications for practice, research and policy are considered.

### 4.1. The dominant barrier landscape: cost, connectivity, training and trust

The four most frequent barrier clusters globally (High Costs (n = 106), Technical Issues (n = 94), Training & Knowledge (n = 91) and Connectivity & Access (n = 89)) together account for roughly one-third of all reported barriers. Their persistence across different health systems suggests that they are a recurrent core of friction in the deployment of telemedicine. Notably, the cost cluster shows the largest barrier-to-facilitator imbalance in the dataset (7.5:1), implying that financial obstacles are extensively diagnosed but rarely matched by validated economic models, reimbursement frameworks or sustainable funding pathways. This is consistent with prior critiques that the business case for telemedicine remains under-specified outside narrow specialty applications [180].

The Privacy & Security and Legal & Regulatory clusters (n = 81 and n = 63 respectively) further refine this picture. Legal-regulatory barriers display a 5:1 deficit relative to facilitators, reinforcing that the gap is not in identifying regulatory friction but in producing transferable governance solutions. In practical terms, telemedicine deployments continue to operate in jurisdictions where licensure portability, cross-border data flows and reimbursement codes lag behind clinical practice [181]. The clustering pattern thus suggests that, even in mature digital ecosystems, telemedicine is currently being scaled on top of legal and financial scaffolding that was not designed for it.

### 4.2. Orphaned barriers: the human and organizational frontier

The systematic identification of “orphaned” barriers, defined as as high-frequency challenges without any documented facilitator counterpart, is the most actionable finding of this review. Psychosocial Factors (n = 46), Psychosocial Support (n = 27), Time & Workload (n = 26), Organizational Issues (n = 26), Privacy Concerns (n = 22), Clinical Limitations (n = 19) and Data Management (n = 18) collectively define a frontier where the field has produced extensive diagnostics but virtually no therapeutics. This negative skew is unlikely to be coincidental.

Several mutually reinforcing mechanisms plausibly contribute. Publication and funding incentives have historically favored the documentation of problems, which are easier to operationalize as research outcomes than solutions, whose effectiveness depends on context-specific co-design and longer follow-up [182]. Implementation studies grounded in established frameworks (e.g., CFIR, RE-AIM) tend to surface organizational and workload barriers but stop short of evaluating organizational interventions to mitigate them. Finally, psychosocial and clinician-burden dimensions sit at the intersection of clinical, behavioral and managerial sciences, and may fall through the disciplinary cracks of conventional telemedicine evaluation. Whatever the mix of causes, the practical effect is the same: managers, clinicians and patients confronted with these barriers cannot, today, draw on a comparable evidence base of facilitators.

### 4.3. The geographic gradient and the digital ladder

Beyond the global picture, the income-stratified analysis reveals a structural gradient in how barriers are organized. High-income countries display diverse, semantically distinct clusters consistent with mature but fragmented digital ecosystems, where Connectivity & Access (n = 82), Financial & Personal Costs (n = 64) and Training & Knowledge (n = 59) coexist with more granular concerns around usability, personal motivation and integration. Upper-middle-income countries occupy an intermediate profile, dominated by Technical Issues (n = 32), Platform Costs (n = 32) and Connectivity & Speed (n = 24). Lower-middle-income countries exhibit only three clusters, in which training, infrastructure, costs and connectivity collapse into compounded thematic blocks.

This gradient empirically substantiates the “digital ladder” hypothesis [183], which argues that infrastructural and foundational prerequisites (electricity, broadband, digital literacy) must be addressed before higher-order enablers such as workflow integration or personalization can deliver measurable benefit. The collapse of conceptual distinctions in lower-resource settings is therefore not analytical noise but a substantive signal: when several barriers are simultaneously binding, they cease to behave as separable problems and instead form a single systemic constraint. This reframing has direct consequences for how telemedicine programs should be designed, sequenced and evaluated in different contexts, and helps explain why models optimized for broadband-rich, highly regulated systems frequently fail to transfer [184]. Because country attribution in this synthesis follows the first author’s first affiliation, this absence is most precisely characterized as an evidence-leadership void: systematic reviews touching on low-income settings exist but are not authored from within them, with consequences for whose questions, framings, and priorities shape the synthesized evidence base.

### 4.4. Asymmetry in the facilitator landscape

The facilitator analysis amplifies the same pattern. High-income countries account for the great majority of documented facilitators (n = 355), upper-middle-income countries report a far smaller and less consistent set (n = 27, ICS often below 0.5), and lower-middle- and low-income countries report none at all. This is not simply a quantitative gap but a qualitative one: it means that, in the contexts where telemedicine is most needed and where implementation conditions differ most sharply from those of high-income settings, there is currently no empirically derived menu of “what works” to inform local program design [185–187]. The risk, well documented in implementation science, is that absence of evidence is interpreted as absence of effective practice, reinforcing a deficit-based narrative that frames lower-income settings as recipients of failed interventions rather than as sites of frugal innovation and reciprocal learning [184].

### 4.5. Methodological contribution

A secondary contribution of this work is methodological. The HDBSCAN-based clustering approach allows large volumes of qualitative findings, drawn from heterogeneous reviews, to be aggregated into reproducible thematic structures. The high internal consistency scores observed for the major clusters (ICS ≥ 0.90 in several cases) support the reliability of the resulting taxonomy, while the open release of data and code enables independent reanalysis, parameter sensitivity testing and extension to other domains of digital health. This addresses a long-standing limitation of narrative syntheses in implementation science, in which classification schemes have been largely investigator-dependent [8–10].

### 4.6. Implications for practice

The findings translate into practice recommendations that are not uniform but conditional on system maturity. In high-income settings, where foundational infrastructure is largely in place, implementation efforts should prioritize the “orphaned” domains identified above: integrating telemedicine into clinician workload calculations, formalizing organizational change management, and developing structured psychosocial support pathways for both patients and providers; furthermore, reimbursement reform, parity legislation and licensure portability remain central given the persistent 5:1 legal-regulatory imbalance [181].

In upper-middle-income settings, where technical issues and platform costs dominate, practice priorities lie in interoperability standards, vendor consolidation and procurement frameworks that prevent the proliferation of fragmented, non-communicating platforms. In lower-middle-income settings, the collapsed cluster structure indicates that telemedicine programs should not be launched in isolation but as part of broader digital health system strengthening, with explicit attention to electricity reliability, last-mile connectivity, frontline worker training and supportive supervision. In low-income settings, where the evidence base is essentially empty, the immediate priority is not large-scale deployment but feasibility studies, formative implementation research and the documentation of context-specific facilitators that can later guide investment [188].

### 4.7. Implications for research

Three research priorities follow directly from the findings. First, the field needs a deliberate shift from barrier documentation to facilitator validation, with funders and journals explicitly rewarding studies that test, rather than describe, implementation strategies. Second, the orphaned-barrier domains should be treated as a coordinated research agenda, including organizational interventions for workload, change-management trials, and structured psychosocial support models, particularly for clinicians, whose burden is poorly captured in current telemedicine outcome sets. Third, locally led implementation research in lower-middle- and low-income countries must be scaled, ideally through partnerships designed as genuine co-production rather than as extractive data collection. The methodology presented here can serve as a living framework, periodically re-run as new reviews accumulate, to monitor whether these gaps are closing.

### 4.8. Implications for policy and global health governance

At the policy level, the results offer concrete guidance for the implementation of the WHO Global Strategy on Digital Health [11] and adjacent regional frameworks. International donors and multilateral agencies (WHO, World Bank, regional development banks) should explicitly earmark research funding for low-income and lower-middle-income settings, and condition large-scale deployment grants on the documentation of locally relevant facilitators rather than barrier inventories alone. Regional bodies such as the African Union’s Africa CDC, the Pan American Health Organization and ASEAN have a particular role in harmonizing digital health regulation across jurisdictions of similar maturity, reducing the duplication of legal-regulatory friction documented in the present cluster analysis.

National governments face context-specific choices. In high-income jurisdictions, the policy frontier is integration: aligning reimbursement, workforce regulation and data governance to remove second-generation frictions. In middle-income jurisdictions, it is consolidation: rationalizing platform ecosystems and codifying interoperability. In lower-resource jurisdictions, it is sequencing: investing in digital and energy infrastructure as health-system foundations rather than as adjuncts to vertical telemedicine programs.

### 4.9. Limitations

This umbrella review has several inherent limitations. As a synthesis of systematic reviews, it inherits any biases present in the included reviews, including publication bias and the over-representation of high-income settings. The HDBSCAN algorithm requires parameter choices that influence cluster granularity; we mitigate this through public release of code and data, but alternative parameterizations may yield finer or coarser thematic structures. Some clusters in lower-resource settings exhibited low internal consistency (ICS < 0.5), reflecting either genuine conceptual heterogeneity or insufficient data for stable pattern recognition. Finally, the World Bank income classification, while standard, is a coarse proxy that may obscure substantial within-country variation in telemedicine readiness, particularly in large federal states. Relatedly, because country attribution relied on the first author’s first affiliation, the evidence absences we report for low-income settings should be interpreted as gaps in research produced from those countries rather than gaps in research about them.

### 4.10. Conclusion

This umbrella review shows that the global telemedicine evidence base does not merely describe digital health inequities but reproduces them: in the volume of evidence available, in the type of barriers that can be analytically distinguished, and in the near-total absence of documented facilitators outside high- and upper-middle-income settings. The 2.6:1 barrier-to-facilitator ratio, the orphaned barrier domains, and the empty evidence space for low-income countries are facets of a single asymmetry. Addressing it requires a coordinated shift, in funding, in methods, and in governance, from documenting why telemedicine fails to systematically generating, in every relevant context, the conditions under which it can succeed. Whether telemedicine narrows or widens the global digital health divide over the coming decade will depend less on the maturity of the technology than on the equity of the evidence base built around it.

## Supporting information

S1 File Methodological analysis details. (DOCX)

S2 File Complete data, all incomes, for barriers and facilitators clusters. (XLSX)

S3 File Complete data, divided by income, for barriers’ clusters. (XLSX)

S4 File Complete data, divided by income, for facilitators’ clusters. (XLSX)

S1 Image Barriers and Facilitators Word-Clouds for High Income derived Instances. (PNG)

S2 Image Barriers and Facilitators Word-Clouds for Upper-Middle Income derived Instances. (PNG)

S3 Image Barriers and Facilitators Word-Clouds for Lower-Middle Income derived Instances. (PNG)

S4 Image Barriers and Facilitators Word-Clouds for Low Income derived Instances. (PNG)

S1 Checklist PRISMA Checklist for the review. (DOCX)
