# Supplementary material for: Mapping global inequities in telemedicine implementation: An umbrella review of barriers and facilitators
Source: PLoS One. 2026 Jul 13;21(7):e0351885. doi: 10.1371/journal.pone.0351885 (PMC13362133; doi:10.1371/journal.pone.0351885)
Supplement: S1 File — (DOCX) [file pone.0351885.s001.docx]

**Data Analysis Details**

The analysis began with the extraction of data from an excel file containing the following columns: Author, Year, Barriers, Facilitators, and Income level. Initially, the “Barriers” and “Facilitators” columns were separated into distinct datasets, and each entry was further segmented into individual items using newline characters to treat every barrier or facilitator as an independent text instance.

Following data acquisition, a comprehensive text preprocessing pipeline was implemented to reduce noise and standardize terminology. All text entries were first normalized by converting them to lowercase and removing extraneous punctuation, leading numbers, bullet-like prefixes, and any parenthetical information, including common abbreviations such as “e.g.” and “i.e.”. Domain-specific standardization was then achieved through a two-stage process. The first stage involved exact matching, wherein a predefined dictionary of synonyms and related phrases was used to replace variations of domain-specific terms with standardized tokens. Subsequently, a fuzzy matching procedure was applied; this method computed similarity scores for n-grams within the text, replacing those phrases that exceeded a predetermined similarity threshold with their corresponding standardized tokens. For example, this method replaced semantically equivalent phrases, such as "the product is difficult to use" and "experienced usability challenges," with a single standardized token like “usability issue” whenever their computed similarity score surpassed a predefined threshold. The text was further processed through lemmatization, reducing words to their base forms, and an extended list of stopwords, encompassing both common English words and domain-specific filler terms, was removed from the tokenized data.

The cleaned and standardized texts were subsequently transformed into numerical vectors using the pre-trained Universal Sentence Encoder from TensorFlow Hub (version 4), which captured the semantic nuances of each text instance. To ensure comparability among the embedding vectors, normalization was applied, and the high-dimensional feature space was reduced using Principal Component Analysis (PCA). This dimensionality reduction retained the most informative components, typically reducing the space to up to 50 components depending on the sample and feature size.

For the core analysis, a density-based clustering approach was employed using the HDBSCAN algorithm. This method identifies natural groupings within data by examining the density of data points. The method excels at discovering clusters of various shapes and densities, which is analogous to identifying distinct patient cohorts that may vary in size and presentation. Critically, it also has a built-in capacity to designate anomalous data points as outliers rather than forcing them into a group, thereby increasing the coherence and potential clinical relevance of the identified clusters. The main design parameter of this algorithm is the minimum number $n_{m}$ of elements needed to compose a cluster. Cluster centroids were computed by averaging the embeddings within each group, and cosine similarity (Intra-Cluster Similarity, ICS) was calculated between these centroids to merge clusters that exhibited a similarity above a predefined threshold $\mu_{s}$. This merging process was implemented via a union-find approach to ensure that semantically related topics were consolidated. Within each cluster, representative keywords were extracted through a term frequency-inverse document frequency (TF-IDF) analysis, and the top-scoring terms were used to generate descriptive labels, with a specific label, “noise,” assigned to the outlier group.

The goal of this core analysis is to obtain clusters that exhibit a high intra-cluster similarity. The HDBSCAN algorithm favors this objective by allowing for a “noise” cluster. However, this may lead to discarding phrases that may have been of interest to identify barriers and facilitators. The trade-off between the consistency of the clusters and the numerosity of the noise was handled using the HDBSCAN parameters $n_{m}$ and $\mu_{s}$. They were determined using a constrained optimization procedure in which these parameters constituted the optimization domain, the optimization function aimed at maximizing the total number of elements out of the “noise” cluster, and the nonlinear constraint function imposed that the median of the ICS was above a given threshold $\sigma_{ic}$($\sigma_{ic}$= 0.5). Moreover, lower and upper bounds were set for $n_{m}$ and $\mu_{s}$ so that the optimization guarantees that the merging of clusters does not jeopardize the nonlinear constraint and that each cluster includes a minimum number of phrases (${n_{m}\in[4, 18], \mu_{s}\in[0.3, 0.99]}$). This optimization procedure provided the optimal values of $n_{m}$ and $\mu_{s}$, namely $\bar{n}_{m}$ and $\bar{\mu}_{s}$, that maximize the number of retained phrases while guaranteeing that each cluster has at least the desired level $\sigma_{ic}$of consistency. Crucially, the outputs of this automated procedure were subjected to qualitative review by two authors (AC and AF) to ensure the resulting clusters were semantically coherent and accurate from a human perspective. This manual verification step informed the final selection of parameters to yield the most interpretable and meaningful results. The optimization was implemented using the SLSQP algorithm.

To further refine the clustering, data points initially classified as noise were isolated and re-clustered using a similar HDBSCAN-based optimization procedure, thereby recovering potential thematic clusters that might have been overlooked in the initial clustering phase.

To examine potential thematic variations across socioeconomic strata, the clustering process was repeated on subsets of data stratified by the Income-Level attribute. By dividing the dataset into distinct income groups and applying the entire methodology, from text cleaning and semantic embedding to clustering and post-processing, this stratified analysis enabled a focused investigation of thematic differences associated with varying income levels.
